# Conservation, Extensive Heterozygosity, and Convergence of Signaling Potential All Indicate a Critical Role for KIR3DL3 in Higher Primates

**DOI:** 10.3389/fimmu.2019.00024

**Published:** 2019-01-28

**Authors:** Laura A. Leaton, Jonathan Shortt, Katherine M. Kichula, Sudan Tao, Neda Nemat-Gorgani, Alexander J. Mentzer, Stephen J. Oppenheimer, Zhihui Deng, Jill A. Hollenbach, Christopher R. Gignoux, Lisbeth A. Guethlein, Peter Parham, Mary Carrington, Paul J. Norman

**Affiliations:** ^1^Division of Biomedical Informatics and Personalized Medicine, University of Colorado, Aurora, CO, United States; ^2^Department of Microbiology & Immunology, University of Colorado, Aurora, CO, United States; ^3^Blood Center of Zhejiang Province, Hangzhou, China; ^4^Department of Structural Biology, Stanford University School of Medicine, Stanford, CA, United States; ^5^Department of Microbiology and Immunology, Stanford University School of Medicine, Stanford, CA, United States; ^6^Wellcome Trust Centre for Human Genetics, and Jenner Institute, University of Oxford, Oxford, United Kingdom; ^7^Institute of Social and Cultural Anthropology, School of Anthropology and Museum Ethnography, University of Oxford, Oxford, United Kingdom; ^8^Immunogenetics Laboratory, Shenzhen Blood Center, Shenzhen, China; ^9^Department of Neurology, University of California, San Francisco, San Francisco, CA, United States; ^10^Basic Science Program, Frederick National Laboratory for Cancer Research, Frederick, MD, United States; ^11^Ragon Institute of the Massachusetts General Hospital, Massachusetts Institute of Technology and Harvard University, Boston, MA, United States

**Keywords:** KIR3DL3, NK cells, KIR, HLA class I, comparative evolution, infectious disease, reproduction

## Abstract

Natural killer (NK) cell functions are modulated by polymorphic killer cell immunoglobulin-like receptors (KIR). Among 13 human *KIR* genes, which vary by presence and copy number, *KIR3DL3* is ubiquitously present in every individual across diverse populations. No ligand or function is known for KIR3DL3, but limited knowledge of expression suggests involvement in reproduction, likely during placentation. With 157 human alleles, KIR3DL3 is also highly polymorphic and we show heterozygosity exceeds that of HLA-B in many populations. The external domains of catarrhine primate KIR3DL3 evolved as a conserved lineage distinct from other KIR. Accordingly, and in contrast to other KIR, we show the focus of natural selection does not correspond exclusively to known ligand binding sites. Instead, a strong signal for diversifying selection occurs in the D1 Ig domain at a site involved in receptor aggregation, which we show is polymorphic in humans worldwide, suggesting differential ability for receptor aggregation. Meanwhile in the cytoplasmic tail, the first of two inhibitory tyrosine motifs (ITIM) is conserved, whereas independent genomic events have mutated the second ITIM of KIR3DL3 alleles in all great apes. Together, these findings suggest that KIR3DL3 binds a conserved ligand, and a function requiring both receptor aggregation and inhibitory signal attenuation. In this model KIR3DL3 resembles other NK cell inhibitory receptors having only one ITIM, which interact with bivalent downstream signaling proteins through dimerization. Due to the extensive conservation across species, selection, and other unusual properties, we consider elucidating the ligand and function of *KIR3DL3* to be a pressing question.

## Introduction

Killer cell immunoglobulin-like receptors (KIR) educate and modulate the cytotoxic and inflammatory activity of natural killer (NK) cells, which target infected and cancerous tissues (1–3). KIR also have a vital role early in placentation, and in the survival and activity of memory T cells (4, 5). The *KIR* genomic locus, in humans comprised of 13 *KIR* genes and two pseudogenes, exemplifies extreme structural, and allelic polymorphism (6, 7). Creating this variation has been duplication, diversification, fusion, and deletion of *KIR* genes (8–10). Suggesting a conserved and essential role for the encoded receptor, *KIR3DL3* is the only gene to have prevailed in all catarrhine primates throughout the rapid evolution of the *KIR* locus (11). Because no ligand or function is known, understanding the evolution and diversity of KIR3DL3 is critical toward understanding its role in human health.

Across geographically and ancestrally diverse human populations, *KIR3DL3* is the only *KIR* gene present in every individual (12). Indeed, of the four genes originally identified as framework for the *KIR* locus (7), only *KIR3DL3* is present on all of the reference haplotypes that have now been characterized (7, 9, 13–15). *KIR3DL3* is also the only gene common to every individual genotyped and every *KIR* locus sequenced from other hominoids (chimpanzee, orangutan, gorilla, gibbon) (16–20) as well as old world monkeys (21–24). In each case, *KIR3DL3* occupies the 5′ terminal of the *KIR* locus, potentially protected from removal during the extensive recombination that creates *KIR* gene content diversity (Figure S1). *KIR3DL3* has multiple alleles, with 120 distinct coding sequences (CDS) identified in humans, and a further 45 identified in total from other catarrhine species (hominoids and old-world monkeys) (18, 25–29). In contrast to other KIR, all of the human *KIR3DL3* alleles encode a full-length protein. Across other catarrhine species, only two chimpanzee *KIR3DL3* alleles have an incomplete reading frame, and these are truncated shortly after the transmembrane domain, potentially producing a secreted form of the protein (19). Together these observations indicate critical requirement for a functioning KIR3DL3 molecule, which is both conserved and polymorphic in all catarrhine primates, and likely under selection to remain that way.

Catarrhine KIR segregate into five broad phylogenetic lineages (8, 30). Of these, lineage I binds non-polymorphic MHC-G molecules, whereas lineage II and III have clearly defined ligands comprising conserved amino acid motifs present on polymorphic MHC-A, -B, or -C subtypes (1, 31). Lineage IV is a branch of lineage II that is specific to monkeys, and has broad MHC class I specificity (16, 32). The ligand binding domains of human KIR3DL3 belong to lineage V, for which no ligand is identified in any species, and the signaling domains belong to lineage III (28). A unifying feature of KIR evolution is that genomic recombination tends to involve complete functional domains, allowing new genes to be formed, whilst others are deleted during large scale gene fusion events (30). This domain and lineage shuffling is possible because *KIR* genes are organized with close correspondence between exons and the encoded functional domains (7). Exons 1 and 2 encode the leader peptide, exon 3-5 encode the Ig domains (D0, D1, D2), exon 6 the stem, and exons 7-9 the transmembrane region and cytoplasmic tail. The Ig domains determine ligand specificity, a brace of ITIMs (immunoreceptor tyrosine-based inhibition motifs) present in the cytoplasmic tail of some KIR molecules mediates NK cell inhibition, and charged residues in the transmembrane of other KIR promote activation through accessory molecules (2, 33, 34). *KIR3DL3* likely encodes an NK cell inhibitory receptor having three Ig domains. Human *KIR3DL3* lacks exon 6, and encodes only the first ITIM (35). All these observations point to a significant impact on ligand binding dynamics and specificity, as well as signaling capability, and suggest KIR3DL3 differs dramatically in function compared with other KIR.

In the NK cells extracted from the blood of healthy human adults, the majority of samples analyzed, KIR3DL3 protein expression is detected rarely, and then at low level (35, 36). Expression is inhibited by methylation of the promoter, and is restored to the cell surface at similar levels to other KIR upon demethylation (37, 38). Promoter and transcription factor sites that are distinct from all other *KIR* explain the unique expression pattern of *KIR3DL3* (39, 40). The fact that expression is switched off in healthy individuals is unusual for KIR, which are randomly acquired by NK cells and expressed irrespective of the presence of their ligand. This suggests unregulated expression of *KIR3DL3* is detrimental. Although the same situation is likely in other apes, where no *KIR3DL3* mRNA has been observed in peripheral blood (8, 16, 20), constitutive expression of lineage V *KIR* is detectable in old world monkeys (41). The majority of humans from whom *KIR3DL3* mRNA has been detected are female, and expression limited to CD56^bright^ NK cells, which are regulatory cells common in the decidua (37). Other KIR mediate transformation of the uterine spiral arteries influencing birth weight and determining risk of pre-eclampsia, fetal growth restriction, or recurrent miscarriage (42–44). The expression profile of *KIR3DL3* thus points to a role in reproduction. The ubiquity, extensive heterozygosity, and potential impact of KIR3DL3 expression on human survival reinforces the need to determine its ligand and function. Here we investigate the functional characteristics of KIR3DL3 through comparative, evolutionary, and population analyses.

## Results

### External Domains of KIR3DL3 Comprise a Unique, Conserved Lineage

**Figure 1:**
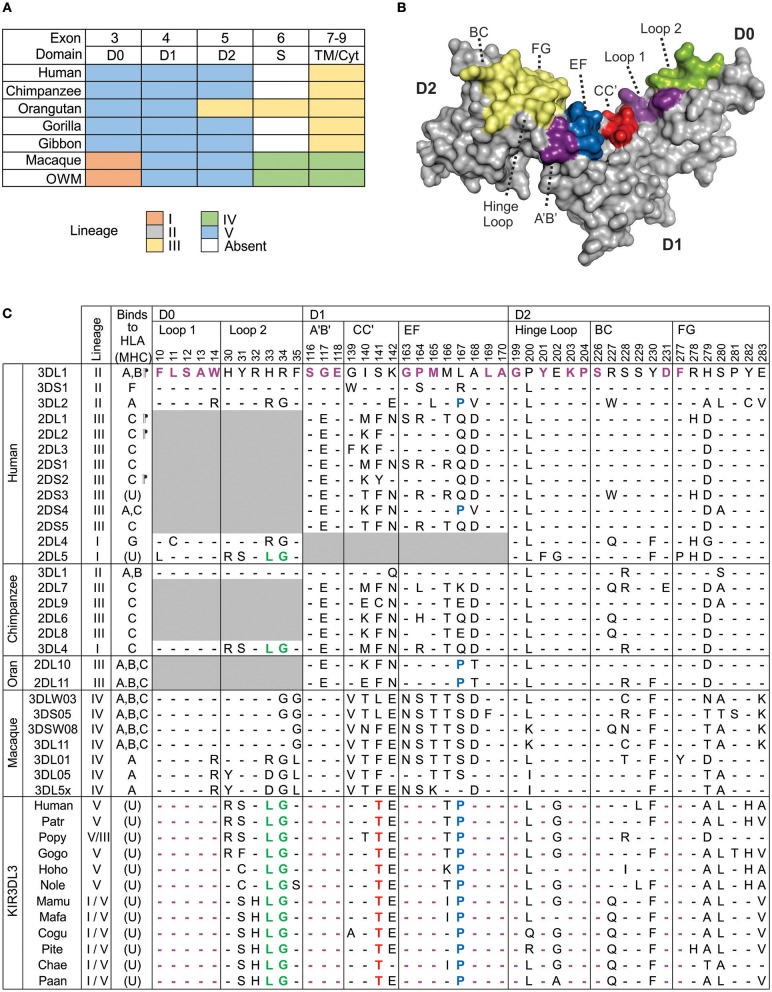
Conserved Ig-like domains of catarrhine KIR3DL3. **(A)** Shown is a summary of the phylogenetic relationships of *KIR3DL3* sequences when compared with other *KIR* sequences from catarrhine primates. Each domain was analyzed separately and colored boxes correspond to the distinct *KIR* lineages; lineages are given in the color key at the bottom. Exon 6, which encodes the stem is absent from all ape *KIR3DL3* except orangutan. Lineage IV is monkey specific (30) and may be part of lineage II (16). OWM—old world monkeys other than macaque. **(B)** Shows a predictive model of the Ig-domains of KIR3DL3. The surface regions corresponding to the HLA class I binding loops of KIR3DL1 are shaded; those containing residues conserved in KIR3DL3 are shaded violet, green, blue, or red to correspond with **(C)**, and others are shaded in yellow. **(C)** Shown are the ligand binding motifs of KIR, as characterized by crystallography. At the left is shown the ancestral KIR lineage, and any known HLA/MHC ligand (A, B, C, F, or G), or unknown (U). (¶) indicates determined by crystal structure (45–48). Top: Residues are numbered according to the mature KIR3DL1 protein. In the alignment underneath, only the amino acid differences from KIR3DL1, which is used as reference, are shown. (-) indicates identity with the reference. Gray boxes indicate domains or residues that are absent from the respective allotype. Below the human KIR, are the respective binding-site motifs from chimpanzee, orangutan, and macaque KIR that are known to bind MHC class I (32, 49–52). The lowest box shows the equivalent motifs from human, other ape, and old-world monkey KIR3DL3; amino acids conserved across all KIR3DL3 molecules are colored red (unique to KIR3DL3), or violet, green, blue (identical to other KIR known to bind MHC, as indicated). Patr, chimpanzee; Popy, orangutan; Gogo, gorilla; Hoho/Nole, gibbon; Mamu, macaque; Pite, red colobus monkey; Cogu, black and white colobus monkey; Chae, African green monkey; Paan, olive baboon.

Domain shuffling by genomic recombination, within and amongst genes belonging to the five ancestral *KIR* lineages, is the most significant mechanism diversifying KIR function (30). The result is that many extant *KIR* genes are comprised from a patchwork representing multiple lineages. To synthesize and extend knowledge of KIR3DL3 lineage ancestry (28, 30), we performed domain-by-domain phylogenetic analysis of catarrhine primate KIR. This analysis included human and gorilla, two species each of chimpanzee, orangutan, gibbon, and macaque, and six further species of old world monkey. For each of the three Ig domains, there is very strong support for the presence of a single phylogenetic group containing the *KIR3DL3*-specific lineage V sequences (Figures S2A–C). In contrast, none of the hominoid species possesses a KIR3DL3 cytoplasmic domain that represents the original lineage V (Figure 1A). In D0 the *KIR3DL3* sequences from all species group as lineage V, except old world monkeys, which group with closely related lineage I (Figure 1A). In D1 and D2, all species analyzed group with lineage V, with exception of orangutans, which group with lineage III in D2. The only other KIR that retains appreciable orthology across these species is KIR2DL4 (KIR lineage I), yet this gene is absent from multiple common haplotypes in several of these species including humans (11). Thus, by comparison with all other KIR molecules (30), the Ig domains of KIR3DL3 are significantly more conserved across catarrhine primates, whereas the cytoplasmic domains are not.

We looked for evidence pointing to the binding partner for KIR3DL3 by comparison with other KIR having established MHC class I ligands. Because residues of KIR involved in ligand interaction remain consistent through all the resolved crystal structures (53), we analyzed the residues of KIR3DL3 that correspond to the eight ligand-binding loops of KIR3DL1 (45) (Figure 1B). Remarkably, 23/45 (51%) residues of KIR3DL3 that comprise these loops, including two complete loops, remain conserved throughout the 23–34 million years of evolution (54) captured by the 14 ape and old world monkey species analyzed (Figure 1C). A further five KIR3DL3 binding-loop residues vary in only one of the species analyzed. None of the variants at any of the 45 binding-loop residues is known to inhibit KIR binding to MHC, with the exception of A-283, which can reduce binding of KIR3DL1 to HLA-B (45), and is present in KIR3DL3 of three species (Figure 1C). Moreover, all except one of the conserved residues and motifs are shared with other KIR that are known to bind MHC. For example, LG33-34 is shared with patr-KIR3DL4, which binds MHC-C (49), and P167 (P71 in lineage III) is shared with human KIR3DL2 and KIR2DS4, modulating their specificity for HLA-A (55). The exception is threonine at position 141, which is unique to KIR3DL3. This position is equivalent to residue 45 of lineage III KIR (e.g., KIR2DL1). Residue 45, which contacts the α-1 helix of the MHC class I molecule, varies across and within KIR lineages, controlling both specificity and avidity of ligand interaction (46, 56, 57). Whereas, threonine at 141 is distinct from any residue at equivalent position in lineage III KIR (Figure 1C), it represents a conservative change from serine that is present in many lineage II molecules, including KIR3DL1. The similarity suggests this amino acid alone is unlikely to prevent binding to MHC. Together, these observations indicate the ligand for KIR3DL3 is likely an MHC class I like molecule; perhaps not a classical highly polymorphic MHC class I, but one highly conserved across catarrhine species. Strongly supporting this argument are the properties of KIR2DL4, which is more divergent in sequence from lineage II and III KIR than is KIR3DL3, yet forms similar structure (58) and retains ability to interact with MHC (31).

A recombination hotspot is located in intron 5 of *KIR3DL3*, between the exons encoding the Ig domains and those encoding the tail (19, 28). As a result, all the hominoid KIR3DL3 molecules include a cytoplasmic tail that originates from lineage III and not lineage V [Figure 1A and (11)]. Moreover, in sharp contrast to the conserved and clear groupings of the lineage V Ig domains, the KIR3DL3 tails fall into at least three sub-clades of lineage III (Figure S2D). In this phylogeny, the most distantly related are the chimpanzee KIR3DL3 tails, which occur on two distinct branches of a group that includes human KIR2DL1-3 and KIR3DL1. Sequences encoding the tails of human, orangutan, gorilla, and gibbon KIR3DL3 form a single group that is related to orangutan KIR2DL10-12 (Figure S2D). Suggesting yet further species-specific diversification, it was not possible to amplify the tail exons of bonobo *KIR3DL3* using primer sequences designed against human or common chimpanzee (20). Finally, the macaque molecule differs from this pattern, sharing ancestry with KIR lineage IV (Figure 1A). These phylogenetic relationships thus demonstrate a complex evolutionary history of the KIR3DL3 cytoplasmic tails with multiple recombination events distinguishing the species analyzed (Figure S2D). To investigate the functional consequences of this dynamic pattern of recombination we examined the ITIM sequences.

### Convergent Removal of an ITIM From Great Ape KIR3DL3

**Figure 2:**
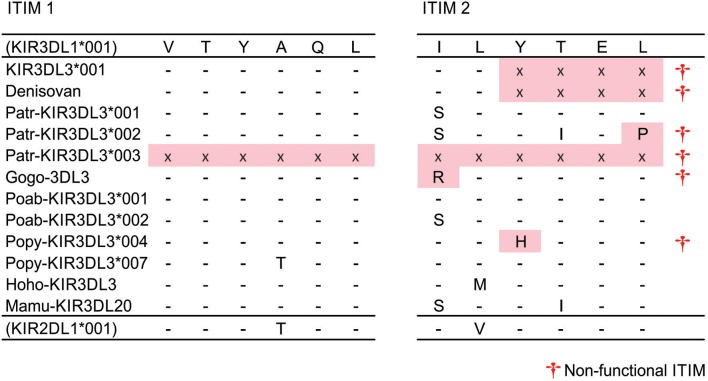
Disruption of second ITIM in great ape KIR3DL3. Shown are the ITIM sequences of KIR3DL3 from multiple species. Only those residues that differ from the reference (human KIR3DL1) are shown. (-) indicates identity with the reference, (x)—residue not present due to early stop codon, (†)—sequence does not conform to the canonical [(I/L/V/S)-Y–(L/V)] ITIM sequence (33) and is likely non-functional. Red shaded boxes indicate mutations that disrupt the ITIM. (+) - disrupted ITIM sequence shared with an allele of another KIR.

Aside from two truncated chimpanzee allotypes that may be secreted (Patr-KIR3DL3^*^003 and ^*^004), the first ITIM is conserved (VTYAQL) across all catarrhine KIR3DL3 molecules (Figure 2). Only one polymorphic residue is present, in Popy-KIR3DL3^*^007, and this matches the polypeptide sequence of human KIR2DL1, which is clearly a strong inhibitory receptor (59, 60). Thus, all of the surface expressed KIR3DL3 molecules possess a first ITIM that is functional. The complete removal of the second ITIM [(I/S)VYTEL] appears to be human specific and mediated by a single nucleotide (A) insertion in the third codon of the motif, creating a translation stop signal (Figure 2). Having the same insertion present in both genomic copies of the archaic human Denisova *KIR3DL3*, but not chimpanzee, indicates this event likely occurred in the hominin lineage (Figure 2). The second ITIM is present and intact in Sumatran orangutan, gibbon, and macaque, but mutated in gorilla, one further chimpanzee and 4/7 Bornean orangutan alleles (Figure 2). Remarkably, all of the mutations that disrupt the second ITIM are different and unique to KIR3DL3. The exception is the ITIM from gorilla KIR3DL3 that shares identity with Gogo-KIR3DLa (a lineage II receptor) through an 86bp segment, which could have been acquired either through gene conversion or through independent mutation (Figure 2). Thus, in every case the second ITIM of KIR3DL3 became disrupted during the time since the tail sequence was acquired from another KIR molecule. That the first ITIM remains intact and multiple (at least 4) independent mutations have disrupted the second, suggests an important requirement for this configuration in all great ape species.

### Diversity of Human KIR3DL3 Focuses to Ig Domains

**Figure 3:**
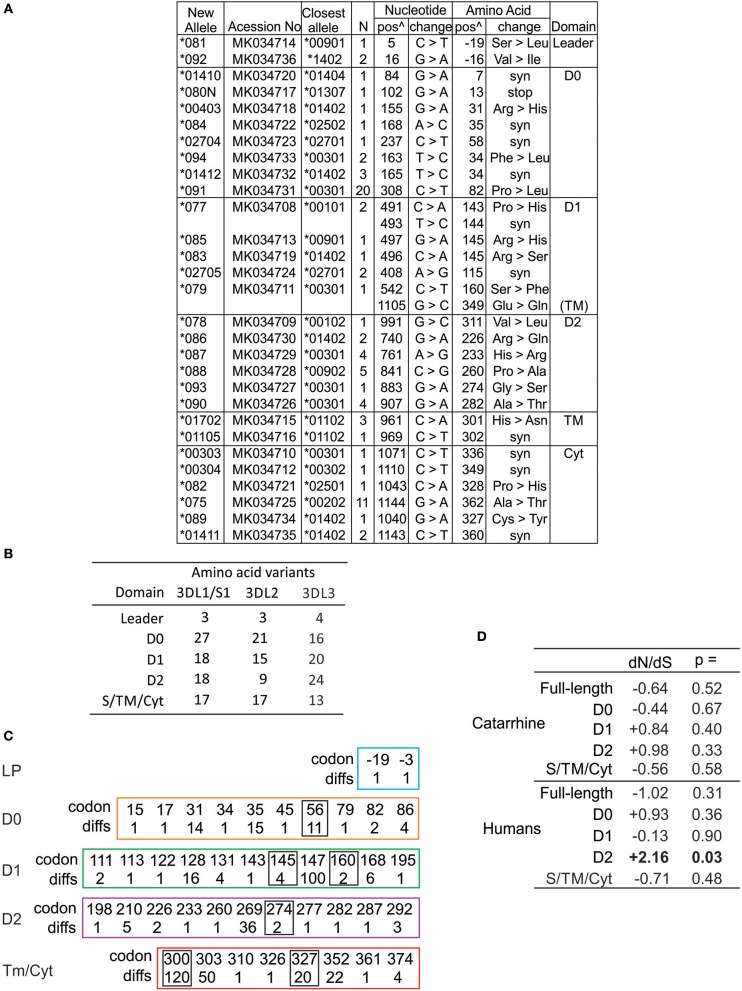
High diversity of human KIR3DL3 Ig domains. **(A)** Shown are the novel *KIR3DL3* alleles characterized for this study from Europeans and Papua New Guineans. From left to right: GenBank ID, the closest matched of the previously established alleles, number of individuals with new allele, nucleotide changes compared to closest match, corresponding amino acid (AA) substitutions, and the domains where the latter occur (Leader–leader peptide, D0-D2–Ig-like domains, TM–transmembrane, Cyt–cytoplasmic). Nucleotides are numbered according to the full length CDS, and amino acids according to the mature protein. (syn) indicates synonymous nucleotide substitution. Additional SNP variants characterized from 1000 Genomes individuals are given in Figure S4. **(B)** Shown is the number of amino acid variants in each of the domains of human KIR3DL1/S1, KIR3DL2, and KIR3DL3. **(C)** Shown are the codon numbers for every amino acid that is polymorphic in KIR3DL3 (codon) and the number of alleles that differ at that position (diffs). Boxes indicate residues having more than one alternative amino acid. The domains are depicted separately. **(D)** Shown are standard dN/dS analyses of *KIR3DL3* nucleotide sequences. Results consistent with (-) negative (purifying) selection or (+) positive (diversifying) selection are indicated, and those statistically significant indicated in bold text. Analysis of catarrhine primate sequences is shown at the top and humans only at the bottom. In each case both the full length CDS and individual domains were analyzed.

The amino acid differences that define human KIR3DL3 variation are shown in Figure S3. In compiling all of the human KIR3DL3 variants, we fully characterized 29 novel alleles (Figure 3A) that had been identified through study of 1,000 individuals from Europe and 200 from Papua New Guinea (61). These alleles represent 19 distinct KIR3DL3 allotypes, nine synonymous variants, and one allele encoding a premature stop codon in D0 (Figure 3A). Together with previously characterized alleles there are 60 amino acid substitutions that characterize the 93 allotypes of KIR3DL3 (Figure S3). To ensure we included similar numbers of individuals and populations as previous analysis of KIR3DL1/S1 and KIR3DL2 (62), we also analyzed data from the 1000 Genomes populations (63), identifying a further 28 SNPs in *KIR3DL3* (Figure S4). By this analysis KIR3DL3 has similar level of polymorphism to KIR3DL2, is less variable than KIR3DL1/S1 (Figure 3B), and more variable than any other KIR (64). Notably, the D1 and D2 of KIR3DL3 contain more coding changes than equivalent domains in KIR3DL1 or 3DL2 (Figure 3B), and four positions in the Ig domains and two in the tail have more than one alternative amino acid (Figure 3C). Comparing the rates of non-synonymous and synonymous mutations showed an elevated rate of non-synonymous mutation in the D2 of human KIR3DL3 (dN/dS = 2.24, *p* < 0.02), likely indicating this Ig domain is evolving under positive selection that favors diversity (Figure 3C). In this broad analysis, none of the other Ig domains showed any statistically significant evidence for selection. Together these observations indicate there is conservation of lineage V function in general across catarrhine primate species, with the exception of humans who appear to be accumulating diversity in the Ig domains of KIR3DL3. The caveat for this finding is that many more human individuals have been examined than any of the other species.

### KIR3DL3 Displays High Heterozygosity

**Figure 4:**
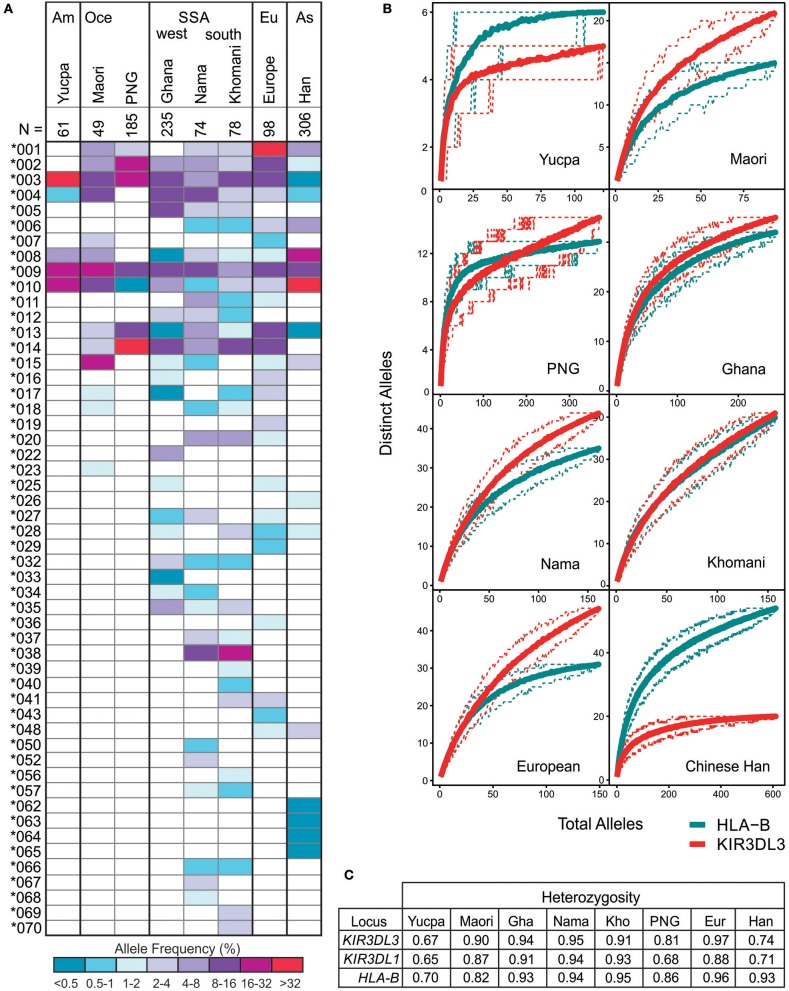
KIR3DL3 shows extensive heterozygosity. **(A)** Shown is the distribution of *KIR3DL3* allotype frequencies from eight human populations analyzed to high resolution. Shades of dark blue to dark red represent the allele frequencies. As given in the key; dark red/purple indicates a higher frequency, dark blue indicates low frequency. Am, South America; Oce, Oceania; SSA, sub-Saharan Africa; Eu, Europe; As, Asia; N, number of individuals. Bold lines group geographically similar populations. **(B)** Shown are rarefaction plots, which compare the distribution of distinct *KIR3DL3* (red) alleles with that of distinct *HLA-B* (blue) alleles in each population. For each gene, the mean number of distinct alleles observed is plotted relative to the number of individuals sampled (solid lines). The 0.05th and 0.95th quantile counts for each gene are also shown (dotted lines). The counts shown are based on 200 independent permutations of resampling. The upper limit of the x-axis is determined by the number of individuals in the population that were genotyped. **(C)** Heterozygosity values for *KIR* and *HLA* alleles in the eight populations.

Including the sequences fully characterized for the current study, there are 157 CDS alleles of human KIR3DL3, encoding 93 distinct polypeptide sequences (Figure S3). To investigate how the multiple KIR3DL3 variants may impact functional diversity across the globe, we analyzed their allele frequencies in representative populations. Focusing on the alleles that encode distinct KIR3DL3 allotypes [denoted by the first three digits (64)], we compared eight populations from six geographical regions that represent major human population groups; West and Southern Africa, South America, East Asia, Europe, and Oceania (Figure 4). Only three KIR3DL3 allotypes (^*^003, ^*^009, and ^*^010) were common to all populations analyzed and a further four (^*^002, ^*^004, ^*^008, and ^*^013) were each found in seven of the eight populations. Indicating an uneven distribution of KIR3DL3 allotypes, the most frequent allotype detected was different for every population. Thus, KIR3DL3^*^001 is the most frequent allele in Europeans, ^*^003 in South American Yucpa, ^*^010 in Chinese Han, ^*^014 in Papua New Guineans, and ^*^038 in Khomani South Africans (Figure 4A). This observation shows KIR3DL3 variation is distributed in a similar manner to HLA, where specific alleles characterize and distinguish human populations (65). Moreover, in all but two of the populations analyzed, the diversity of *KIR3DL3*, as measured by the mean number of distinct alleles per individual (Figure 4B) and heterozygosity (Figure 4C and Figure S5), equaled or exceeded that of *HLA-B*. The two exceptions were populations representing South Americans and East Asians, where natural selection favoring the increased frequency of specific *KIR A* haplotypes has been demonstrated (66, 67), thereby reducing the heterozygosity of KIR3DL3.

### Positive Selection Favors Aggregation of Human KIR3DL3 Molecules

**Figure 5:**
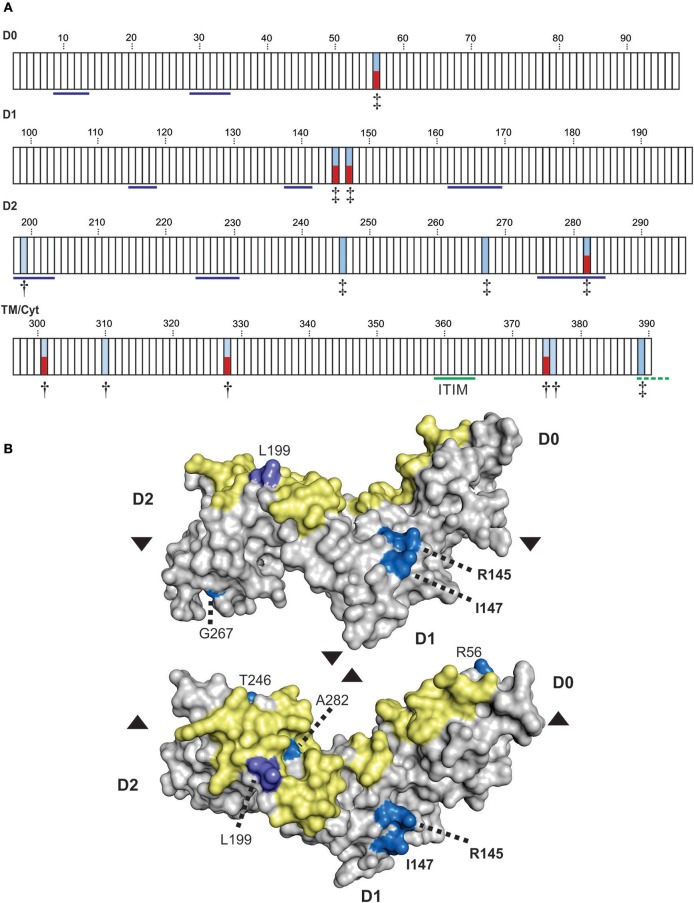
Positive selection diversifies residues of KIR3DL3 involved in protein aggregation. **(A)** Shown are the results from codon-by-codon selection analysis of KIR3DL3. Each box corresponds to an amino acid residue of human KIR3DL3, numbered according to the mature protein. Blue shading corresponds to positively selected sites (^†^*P* > 0.9, ^‡^*P* > 0.95). Red shading indicates those positively selected sites that are also polymorphic in humans. Residues underlined in blue in D0-D2 correspond to the HLA class I binding sites of KIR3DL1 (45). The functional immunoreceptor tyrosine-based inhibitory motif (ITIM) is underlined in green, the non-functional second ITIM, which is interrupted by a stop codon in human KIR3DL3, is underlined in dashed green. The evidence for selection in each domain is supported by a posterior probability of >0.90. The four domains (D0, D1, D2, and TM/Cyt) were analyzed separately. **(B)** A space-filling model of the Ig-domains of KIR3DL3. Positively selected residues are denoted by shades of blue- bright blue: PP > 0.95, light purple: PP > 0.90. The region corresponding to the HLA class I binding site of KIR3DL1 is shaded in yellow. The amino acid residues are numbered as **(A)**. Upper–“side” view, lower–“top” view; where arrows indicate the relative rotation.

To refine the analysis of KIR3DL3 evolution we used a codon-based test for selection, which analyzes the rate of amino acid changes at individual residues through phylogenetic comparisons (68). To account for the extensive recombination characteristic of *KIR* evolution we analyzed each domain independently. This analysis identified 13 amino acid residues under selection for diversity in hominoids, seven in the Ig domains and six in the tail (Figure 5A). Seven of these sites (four Ig, three tail) are polymorphic in humans. Residue 282, which occurs in D2, is both positively selected for diversity and polymorphic in humans and corresponds with the HLA binding region of KIR3DL1, as determined from crystal structure [(45) and Figure 1]. In analyzing KIR3DL1, residue 282 has potential to impact ligand binding strength and specificity (69, 70). However, in contrast to KIR3DL1, where the positively selected polymorphic sites focus almost exclusively to the HLA binding region, diversifying selection on KIR3DL3 does not appear to have been focused exclusively toward ligand binding. Instead, there are two positively selected polymorphic residues (both PP > 0.95) in D1 that do not correlate with known KIR-ligand binding sites. Homology modeling of KIR3DL3 tertiary structure showed these residues, 145 (R/H/S) and 147 (I/V), occur on the side of the molecule (Figure 5B) and potentially orthogonal to the cell membrane. Residue 145 is equivalent to residue 50 of lineage III KIR, which has the same alternative amino acids (R/H/S) also maintained through diversifying selection (17). Importantly, these residues likely mediate aggregation or polymerization of KIR molecules (71).

**Figure 6:**
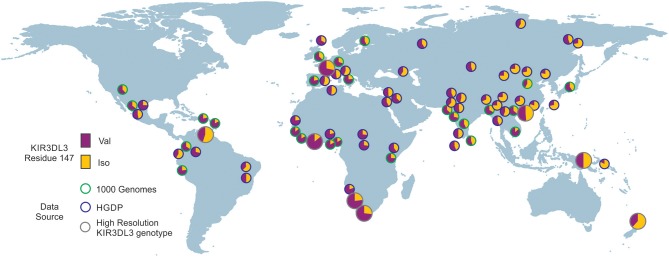
Residue 147 of KIR3DL3 is heterozygous in all human populations. Shows the frequency distribution of dimorphism at KIR3DL3 residue 147. The ancestral allele (valine) is indicated in purple and the derived (isoleucine) in gold. The eight populations analyzed to high resolution are indicated by larger circles having gray rings. Green rings—frequencies obtained from 1000 genomes exome data. Blue rings—frequencies obtained from HGDP data (SNP rs270790 a/g).

To analyze the impact of KIR3DL3 residues 145 and 147 variation on worldwide KIR diversity we examined their polymorphism within alleles and populations. Comparison with other ape sequences revealed R145 and V147 are likely the respective ancestral hominid alleles (Figure S6). Residue 145 has a derived substitution in four human allotypes (^*^037, ^*^046, ^*^083, and ^*^085; Figure S3). These alleles are at low frequency or absent from the eight populations studied (Figure 4A) and the residue is not polymorphic within any of the other ape species (Figure S6). In contrast, residue 147 has the derived allele in 100/157 allotypes, is polymorphic in all eight populations, and the I/V polymorphism is shared with chimpanzee and humans (Figure 3 and Figure S6). The latter observation is very good evidence for trans-species polymorphism, and thus a strong indicator that balancing selection is acting on KIR3DL3. Thus, in this analysis, residue 147 is seen as having more significant impact than residue 145 on worldwide NK receptor diversity. Expanding the study shows both the isoleucine and valine forms of KIR3DL3 residue 147 are present in every one of a further 80 human populations (Figure 6). Thus, although populations are highly differentiated in KIR3DL3 allele spectra (Figure 4), every human population retains dimorphism at residue 147 (Figure 6).

## Discussion

KIR3DL3 is highly polymorphic, and unusually for KIR, present in every human individual and conserved across catarrhine primate species. Although this ubiquity indicates a critical role in survival, no ligand has been identified and no function or expression pattern fully characterized. Through this analysis, we set out to investigate by comparative genomic studies if there is any evidence from the molecular evolution of KIR3DL3 that provides clues to its function.

We examined all known CDS alleles of KIR3DL3, characterized additional alleles and identified any further SNP variants present in the 1000 Genomes sequence data. In this extensive survey we identified only one human allele of *KIR3DL3* having a premature stop codon, thus unlikely to be expressed, and this was observed in a single heterozygous individual (Figure 3A). This is the only *KIR3DL3* allele, of those characterized from >3,000 individuals analyzed at similar high resolution that does not code a full-length polypeptide sequence (25–27, 62, 66, 72–74). In this regard, *KIR3DL3* differs from all other *KIR*, which either have multiple non-expressed alleles, show presence/absence polymorphism, or both. Although previous genotyping analysis has identified *KIR3DL3* in every catarrhine individual examined, segregation analysis implied a small number of macaque haplotypes lacked the gene (75). That the macaque data was acquired by PCR genotyping and not shotgun sequencing, and the absence was characteristic of specific haplotypes, raises the possibility that one or more alleles were present but not detected by virtue of the PCR primer sequences. Thus, *KIR3DL3* is potentially present as an open reading frame in every hominoid and old-world monkey. These findings strongly reinforce the view that possession of functional KIR3DL3 is essential for survival. Moreover, although the *KIR* loci of platyrrhine and catarrhine primates diverged independently, KIR having a similar configuration of three Ig domains, and no stem have been detected in new-world monkeys (76), suggesting this requirement could extend to all higher primates.

One striking consequence of the large number of KIR3DL3 alleles is very high heterozygosity, rivaling that of HLA-B in the human populations studied, and likely replicated in other catarrhine species (11, 23, 24, 75). Although HLA-B is under strong selection to maintain sequence diversity (77, 78), it has never been fully established whether this is due to a frequency-dependent mechanism, heterozygous advantage or both. For KIR3DL3 we determined first that two sites in the Ig domain of the molecule likely involved in receptor aggregation are under selection for diversity across hominoid species, and secondly that in humans, heterozygosity in every population is focused to one of these sites. Although I-147 and V-147 are each represented by multiple full-length *KIR3DL3* alleles, these findings show that dimorphism at this site is driving the high heterozygosity of human KIR3DL3. Together with their relatively even frequencies across populations, this is more consistent with a heterozygous advantage model of balancing selection, than with frequency-dependence. This raises the intriguing possibility that KIR3DL3 dimers or multimers could develop most efficiently when both alleles are present. *KIR A* and *B* are two forms of *KIR* gene content haplotype that are also maintained in all human populations by balancing selection (6, 66). The three KIR3DL3 allotypes that we show here are common to all populations represent this dichotomy, KIR3DL3^*^009 and ^*^010 are characteristic to *KIR A* haplotypes, and KIR3DL3^*^003 characterizes *KIR B* haplotypes (13). That all three of these common allotypes have isoleucine at position 147 shows clearly that the requirement for variation at this position in all human populations is independent of the requirement to retain *KIR A* and *B* haplotypes.

Whereas we found significant sequence variation and heterozygosity of KIR3DL3, there is remarkable conservation in the residues corresponding to those of other KIR molecules that are involved in ligand binding. This leads us to propose the same residues, or binding loops, mediate KIR3DL3 interaction with ligand. The observed mixture of conserved residues and conservative changes at key sites supports the model that KIR3DL3 binds a ligand of very similar structure to classical MHC class I, but with limited polymorphism and cross-species variation. A parallel can be drawn with KIR2DL4, which is the only other KIR molecule retaining significant orthology across hominoids (11) and interacts with MHC-G (31). In contrast to *KIR3DL3*, the *KIR2DL4* gene is absent from multiple common haplotypes in humans and other hominoid species (11), and the most prominent, trans-species, polymorphism determines whether or not the receptor is expressed at the cell surface (79). In gibbons, there is no MHC-G, and few functional forms of KIR2DL4 are present (17), whereas macaques possess a likely MHC-G precursor, concurrent with greater frequency of functional KIR2DL4 (76, 80). Thus, the evolution of KIR2DL4 correlates with the evolution of moderately polymorphic MHC-G (17). Likewise, evolution and diversity of lineage III KIR correlates with emergence of MHC-C (49), and in old world monkeys, expansion of lineage II KIR corresponds with expansion in number of the *MHC-A* and -*B* genes (81). The ligand binding domains (and gene presence) of KIR3DL3 have remained conserved for a minimum of 23–34 million years, since the hominoid and old world monkey split (54). The search for a ligand must therefore include MHC molecules also conserved among these species such as CD1d or MR1, which have established roles in innate immunity (82). Of note, both CD1d and MR1 are expressed by fetal cells having potential to contact maternal NKT cells in the placenta during pregnancy. CD1d is expressed by fetal trophoblasts, and may be co-expressed with HLA-G, and MR1 is expressed by fetal macrophages (83, 84).

KIR clustering is important for inhibition because it helps both ligand binding and downstream signaling (36, 85). We identified five positively selected sites in the transmembrane/cytoplasmic tail region, three of which vary in humans. The implication of this finding is unknown. These polymorphisms could also affect receptor aggregation, positioning of the tail within the membrane or cytoplasm, or secondary structure coiling, which is essential for accessory molecule binding (36). KIR inhibit NK cell responses by blocking activation signals at the source, requiring their placement in, or near an immune synapse (2). Activation signals are blocked through interaction of the ITIMs with bivalent SHP-1 (Src homology region 2-containing protein tyrosine phosphatase-1) or SHP-2 (86, 87). Enabling this interaction, lineage II and III KIR (e.g., KIR2DL1 and KIR3DL1) each possess two cytoplasmic tail ITIMs separated by ~25 amino acid residues (2). In contrast, Ly49 receptors, which are the functional equivalents of KIR in mice, possess only one ITIM but form dimers that are able interact with SHP molecules (33). Dimerization brings the two ITIMs into proximity allowing interaction with SHP. Thus, the evidence we have obtained points to a similar mode for KIR3DL3 as a functional inhibiting receptor. The first ITIM remains intact and conserved across species and human alleles, as do the two serine and one tyrosine residues (S364, S394, and T399: not shown) shared with KIR3DL1 that enhance receptor phosphorylation (88). Convergent removal of the second ITIM across species combined with selection at sites known to mediate receptor aggregation strongly indicate that dimerization of KIR3DL3 and signaling through SHP are intricately related. Supporting this hypothesis are the suggestion that lineage V represent an ancestral form of KIR, and the close relationship of KIR3DL3 with KIR2DL5 [(16, 28, 30) and Figure S2]. KIR2DL5 also has only one standard ITIM and is an SHP-2 recruiting inhibitory receptor (89, 90).

Multiple other KIR have been investigated due to their known interaction with HLA-class I molecules, or their association with disease based on presence/absence polymorphism. Because high-resolution studies of KIR are limited, and KIR3DL3 is always present, no disease association study has detected any signal for KIR3DL3. Here we present indirect evidence that KIR3DL3 polymorphism has a function critical for human survival, and identify a site in the Ig domain worthy of our further focus in disease and functional studies. We also show that the mode of KIR3DL3-mediated inhibition is likely conserved across hominoids, reflecting an ancestral KIR, and that the ligand is also likely conserved through a deeper time scale, including all catarrhine primates. Given the properties defined herein for KIR3DL3, as well as previous examples of expression limited to uterine NK cells, a role in reproduction seems the most likely candidate function for KIR3DL3.

## Materials and Methods

### *KIR3DL3* Sequence Data Set

For the purposes of this study we considered only those sequences that are distinguished in the exons (code determining sequence: CDS) as distinct alleles. Other sequence differences are discussed where relevant. Sequences corresponding to all known CDS alleles of human *KIR3DL3* were obtained from the Immuno Polymorphism Database (IPD) release 2.7.1 (64), and from other species as listed. We used sequences from *Pan troglodytes* (Patr) and *Pan paniscus* (Papa) chimpanzee (20), *Gorilla gorilla* (Gogo) Gorilla (30), Bornean (*Pongo pygmaeus*: Popy) and Sumatran (*Pongo abelii*: Poab) orangutans (16, 18, 91), eastern hoolock (*Hoolock hoolock leuconedys:* Hoho) and northern white cheeked (*Nomascus leucogenys:* Nole) gibbons (19), Rhesus (*Macaca mulatta:* Mamu) and Crab-eating (*Macaca fascicularis*:Mafa) macaques, African green monkey (*Chlorocebus aethiops*: Chae), Black-and-white Colobus monkey (*Colobus guereza*: cogu), Olive baboon (*Papio anubis:* paan) (24), and Red colobus monkey (*Piliocolobus tephrosceles*: Pite) (92). The sequences are named in accordance with the updated non-human primate KIR nomenclature database (IPD-NHKIR) release 1.1.0.1 (93); all are termed *KIR3DL3*, except for old-world monkeys, which are represented by *KIR3DL20* (93). Archaic human *KIR3DL3* sequences were extracted from the high (30x) coverage Denisovan genome (94) using the bioinformatics pipeline described (62) and adapted for non-paired reads. The human KIR3DL3^*^00101 sequence was used as the reference. Previous names for human KIR3DL3 include KIRC1, KIR3DL7, and KIR44.

### Newly-Identified Alleles

In addition to 120 human *KIR3DL3* CDS alleles obtained from the IPD, we included nine identified from southern African KhoeSan (25), and 29 identified during the 17th International HLA and Immunogenetics Workshop (IHIW) (61). The latter represent alleles identified from European (*N* = 1,000) and Papua New Guinean (*N* = 200) subjects, using high throughput sequencing (62). For the current study, these alleles were confirmed by Sanger sequencing from independent PCR amplifications, using primers, and methods as described previously (73). These novel allele sequences were deposited to GenBank and the IPD.

#### Novel SNPs Identified in 1000 Genomes Data

We used whole-exome data of 2,112 individuals from the May 2013 release of the 1000 genomes (63). All read-pairs that mapped within coordinates chr19:55228188-55383188 and chr19_gl000209_random of the human reference genome build hg19 were extracted. The reads were analyzed using the PING pipeline (62) with KIR3DL3^*^00101 as the reference allele. Novel coding region SNPs identified in this manner were validated by generating an independent alignment of sequence reads, and manual inspection, as described (62). Novel SNPs were defined as those not present in IPD release 2.7.1.

### Molecular Analysis

#### dn/dS

Standard dN/dS analysis with the *Z*-test for selection, was performed using Mega 6.0 (95). The hypothesis of neutral evolution was tested using the Kumar method (95), supported by 1,000 bootstraps.

#### Phylogenetic Analysis

Neighbor-joining phylogenic analyses of the full CDS and each functional domain were generated using the Tamura-Nei model, with pairwise deletion, using Mega 6.0 (95). All trees are shown with support from 1,000 bootstraps. We selected 3–5 alleles representative of the allelic diversity of each species.

#### Bayesian Selection Analysis

Codon-by-codon Bayesian analysis were performed using the Datamonkey Fast Unconstrained, Bayesian AppRoximation (FUBAR) method (68). Each domain was analyzed separately, and only included alleles unique in that region. Sequences were trimmed of stop codons. For D0-D1, only *KIR* lineage V sequences were used.

#### Structural Modeling

The 3D structure model of KIR3DL3^*^001 Ig domains was generated by homology modeling using Swissmodel, https://swissmodel.expasy.org/ (96).

### Population Genetic Analysis

Frequencies of *KIR* and *HLA class I* alleles were obtained from the following populations: Yucpa Amerindians (66), Maori (72), sub-Saharan Africans: Ga-Adangbe from Ghana (73), and Nama and Khomani San from southern Africa (25), Europeans and Papua New Guineans (61), and Chinese Southern Han [(97–99); Deng et al., unpublished]. Heterozygosity was calculated according to Nei and Tajima (100) using the allele frequencies within each population. Rarefaction curves were calculated with a custom python script that randomly samples *n* alleles from each population where *n* is each integer from 1 to the total number of genotyped alleles in the population. We report the mean count of unique alleles, as well as the 0.05 and 0.95 quantile of 200 replicates at each *n*.

#### Analysis of Amino Acid Residue 147

For the 1000 genomes data, SNP calls from nucleotide 503 in the full length CDS of *KIR3DL3* were generated using PING (62). Following filtering for *KIR3DL3* specific sequence reads, only those individuals having read depth >20 at position 503 were used (*N* = 1,473: representing 28 populations). Human Genome Diversity Panel (HGDP) data (101) was obtained from 889 individuals representing 52 populations, who had been whole-genome SNP genotyped, and available at ftp://ftp.cephb.fr/hgdp_supp15/. Genotype calls from CDS position 503 of *KIR3DL3* (dbSNP ID: rs270790) were extracted using PLINK (102). For populations duplicated across the two data sets, only the 1000 genomes data were used.

## Author Contributions

PN and LL conceived and designed the experiments. LL, PN, JS, KK, ST, and CG analyzed data. KK, NN-G, and ST performed lab experiments. ZD and LG provided new data. JH, AM, SO, MC, and PP provided materials. PN and LL wrote the paper, and all authors approved the final submitted version.

### Conflict of Interest Statement

CG owns stock in 23andMe, Inc. The remaining authors declare that the research was conducted in the absence of any commercial or financial relationships that could be construed as a potential conflict of interest.

## Supplementary Material

Figure S1 Genomic organization of KIR. **(A)** Shown is a map of the human *KIR* locus. Boxes indicate genes. White circles indicate nodes of frequent recombination, and the lines indicate common haplotypes. **(B)** Summarizes the domain organization of human KIR in relation to the exon structure of the genes. Hatched boxes indicate pseudo-exons. LP, leader; D0-D2, domains 0–2; S, stem; TM, transmembrane; Cyt, cytoplasmic tail. Green X indicates ITIM, red X indicates charged residue that allows association with DAP12, which contains activating motifs.

Figure S2 Phylogenic analysis. Shown is domain-by-domain phylogenetic analysis of nucleotide sequences comprising catarrhine KIR. **(A)** D0, **(B)** D1, **(C)** D2, **(D)** S/TM/CYT. Colored boxes indicate the groupings of each lineage: I, orange; II, gray; III, yellow; IV, green; V, blue. Papa and Patr, chimpanzees; Poab and Popy, orangutans; Gogo, gorilla; Hoho and Nole, gibbons; Mamu and Mafa, macaques; Pite and Cogu, colubus monkeys; Chae, African green monkey; Paan, olive baboon. If no prefix before KIR: human. Neighbor-joining trees are shown with support from 1,000 bootstraps, only nodes above 50% are shown. Triangles indicate branches that were collapsed for clarity. Exon 6 (stem) has a hominoid-specific deletion (16). **(D)** This phylogeny supports the possibility that all the hominoid KIR3DL3 cytoplasmic tails originated from a single recombination event that removed the stem whilst introducing a lineage III tail. In this case the chimpanzee molecules then diverged through subsequent recombination and the orangutans regained the stem sequence during a different recombination that involved exon 5, converting D2 to lineage III. The old world monkey tails are homogenized amongst all the KIR molecules (lineage IV), and it is not possible to determine whether or not the tails of KIR3DL3 represent the ancestral lineage V.

Figure S3 The amino acid residues that distinguish human KIR3DL3 allotypes. Shown is an alignment of the amino acid differences that define the allotypes of human KIR3DL3. Only differences are shown. Dashes indicate identity with the reference sequence (KIR3DL3^*^001). Amino acid residues are numbered according to the mature protein. LP, leader peptide; D0, Ig-like domain 0; D1, Ig-like domain 1; D2, Ig-like domain 2; Tail, transmembrane and cytoplasmic regions.

Figure S4 SNP variations of KIRDL3 identified in the 1000 genomes data. Shown are SNPs identified by analyzing the exome data of 2,112 individuals from the 1000 genomes project. The novel SNPs (i.e., not present in IPD release 2.7.1) were identified by filtering sequence reads to be specific for *KIR3DL3* and aligning to *KIR3DL3*^*^*00101* as a reference sequence. From left to right: Population where SNP was found, number of individuals with new allele, novel nucleotide changes relative to *KIR3DL3*^*^*00101*, corresponding amino acid (AA) substitutions, and the domains where the latter occur (LP, leader peptide; D0-D2, Ig-like domains; TM, transmembrane; Cyt, cytoplasmic). Nucleotides are numbered according to the full length CDS, and amino acids according to the mature protein. (syn) indicates synonymous nucleotide substitution. The populations are named according to the 1000 genomes project; ACB, African Caribbean in Barbados; CDX, Chinese Dai in Xishuangbanna, China; CHB, Chinese Han in Beijing; CHS, Chinese Southern Han; ESN, Esan in Nigeria; GIH, Gujarati India in Texas; GWD, Gambian; IBS, Iberians in Spain; ITU, Indian Telugu; JPT, Japanese; KHV, Kinh in Ho Chi Minh City, Vietnam; LWK, Luhya in Kenya; MSL, Mende in Sierra Leone; PEL, Peruvian in Lima, Peru; PJL, Punjabi from Lahore, Pakistan; STU, Sri Lankan Tamil; TSI, Tuscans in Italy.

Figure S5 Fst and heterozygosity values. Heterozygosity of *KIR* and *HLA class I* alleles per population.

Figure S6 Codon-by-codon selection analysis. **(A)** Shown are the sites identified using codon-by-codon selection analysis of *KIR3DL3*. Each domain was analyzed individually. Center column shows the posterior probability obtained using FUBAR; light blue boxes correspond to *P* > 0.90, dark blue *P* > 0.95. At the right is shown the reference amino acid residue (AA) and the variants across the species analyzed (Var). Site numbers of amino acids are in reference to the mature protein. **(B)** Shown are the variants present at residues 145 and 147 of KIR3DL3 in ape species. ^*^ -identified in one 1000 genomes sample and not included in selection analysis (Figure S4).

## Abbreviations

KIR: Natural killer cell immunoglobulin-like receptor
D0, D1, D2, S/TM/CYT: Domains 0-2, stem, transmembrane, cytoplasmic regions encoding the tail
NK cell: Natural killer cell.
