# Supplementary material for: Conservation, Extensive Heterozygosity, and Convergence of Signaling Potential All Indicate a Critical Role for KIR3DL3 in Higher Primates
Source: Front Immunol. 2019 Jan 28;10:24. doi: 10.3389/fimmu.2019.00024 (PMC6360152; doi:10.3389/fimmu.2019.00024)
Supplement: Figure S1 — Genomic organization of KIR. (A) Shown is a map of the human KIR locus. Boxes indicate genes. White circles indicate nodes of frequent recombination, and the lines indicate common haplotypes. (B) Summarizes the domain organization of human KIR in relation to the exon structure of the genes. Hatched boxes indicate pseudo-exons. LP, leader; D0-D2, domains 0–2; S, stem; TM, transmembrane; Cyt, cytoplasmic tail. Green X indicates ITIM, red X indicates charged residue that allows association with DAP12, which contains activating motifs. [file Data_Sheet_1.PDF]

A

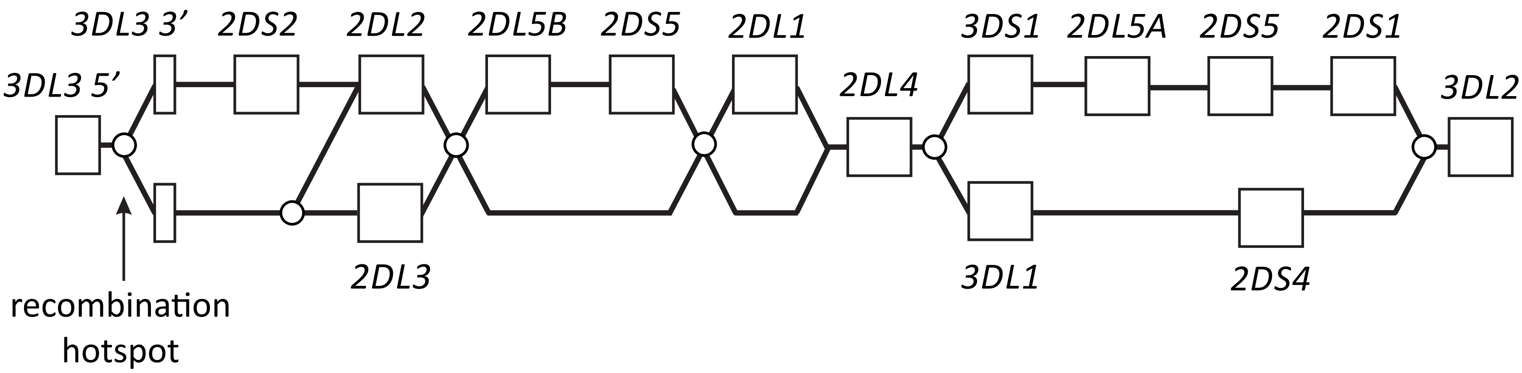
3DL3 5'
3DL3 3'
2DS2
2DL2
2DL5B
2DS5
2DL1
2DL4
3DS1
2DL5A
2DS5
2DS1
3DL2
2DL3
3DL1
2DS4
recombination hotspot

B

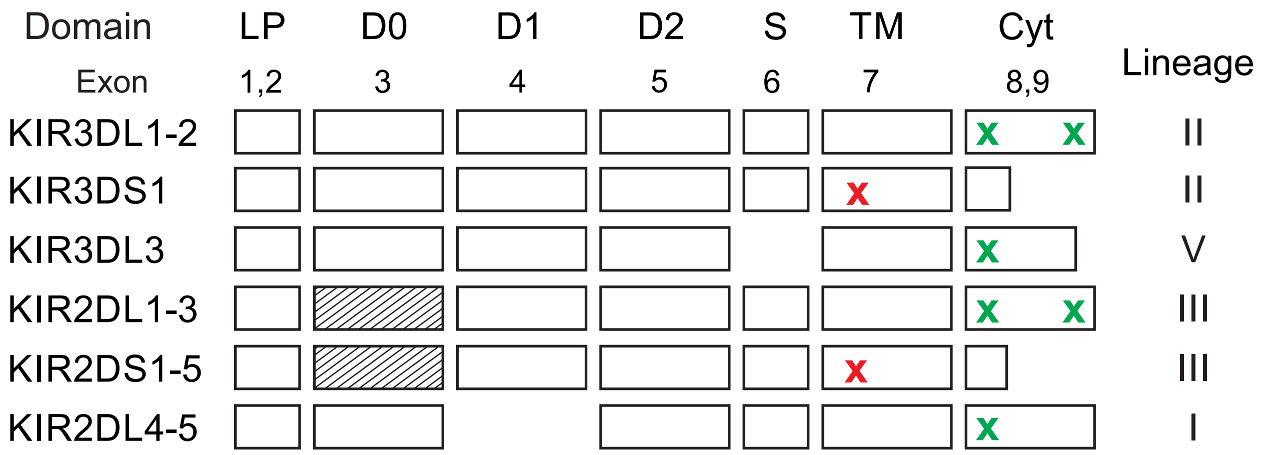
Domain
LP
D0
D1
D2
S
TM
Cyt
Lineage
Exon
1,2
3
4
5
6
7
8,9
KIR3DL1-2
II
KIR3DS1
II
KIR3DL3
V
KIR2DL1-3
III
KIR2DS1-5
III
KIR2DL4-5
I

Fig. S1

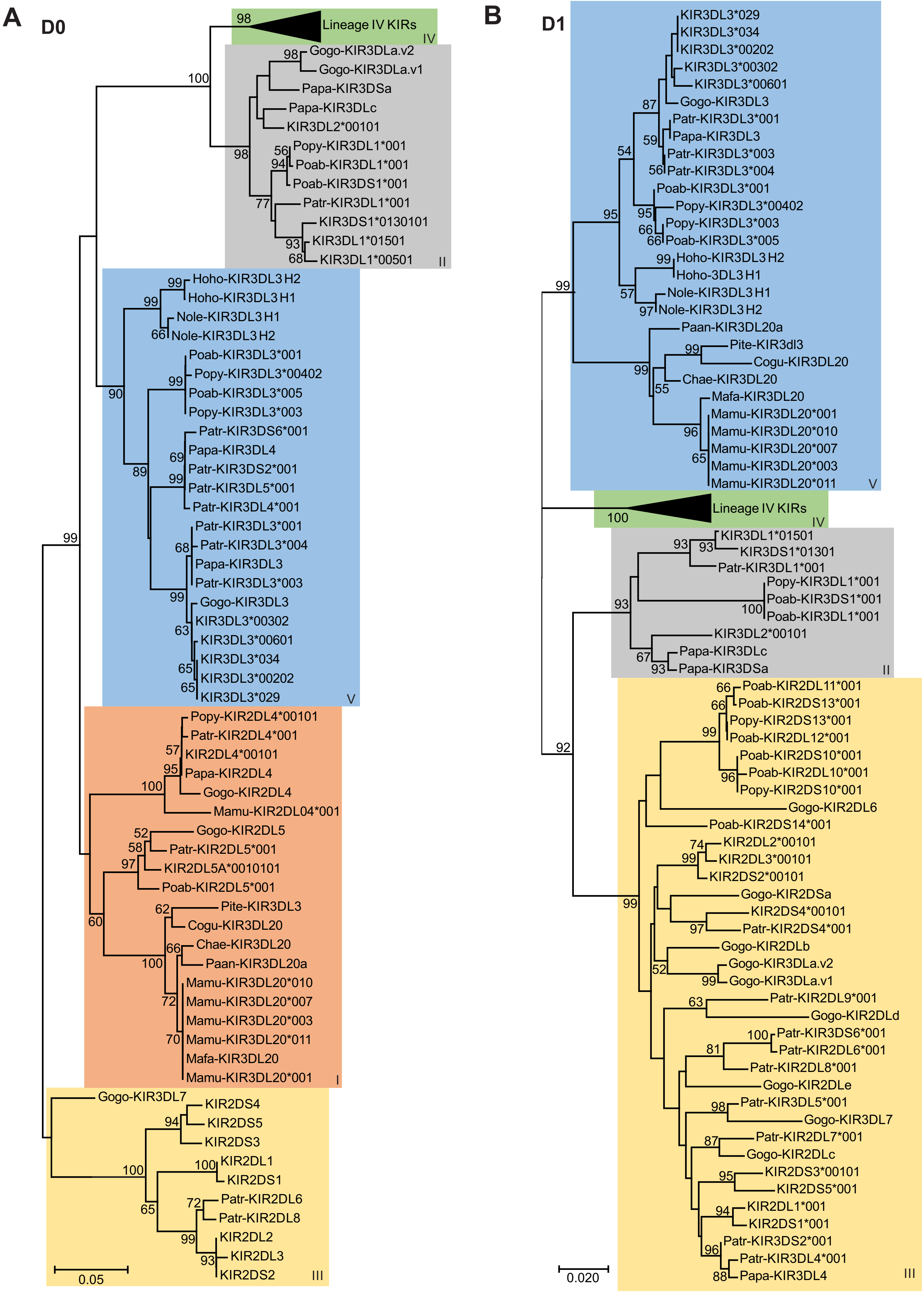
A
D0
Lineage IV KIRs
IV
Gogo-KIR3DLa.v2
Gogo-KIR3DLa.v1
Papa-KIR3DSa
Papa-KIR3DLc
KIR3DL2*00101
Popy-KIR3DL1*001
Poab-KIR3DL1*001
Poab-KIR3DS1*001
Patr-KIR3DL1*001
KIR3DS1*0130101
KIR3DL1*01501
KIR3DL1*00501
II
Hoho-KIR3DL3 H2
Hoho-KIR3DL3 H1
Nole-KIR3DL3 H1
Nole-KIR3DL3 H2
Poab-KIR3DL3*001
Popy-KIR3DL3*00402
Poab-KIR3DL3*005
Popy-KIR3DL3*003
Patr-KIR3DS6*001
Papa-KIR3DL4
Patr-KIR3DS2*001
Patr-KIR3DL5*001
Patr-KIR3DL4*001
Patr-KIR3DL3*001
Patr-KIR3DL3*004
Papa-KIR3DL3
Patr-KIR3DL3*003
Gogo-KIR3DL3
KIR3DL3*00302
KIR3DL3*00601
KIR3DL3*034
KIR3DL3*00202
KIR3DL3*029
V
Popy-KIR2DL4*00101
Patr-KIR2DL4*001
KIR2DL4*00101
Papa-KIR2DL4
Gogo-KIR2DL4
Mamu-KIR2DL04*001
Gogo-KIR2DL5
Patr-KIR2DL5*001
KIR2DL5A*0010101
Poab-KIR2DL5*001
Pite-KIR3DL3
Cogu-KIR3DL20
Chae-KIR3DL20
Paan-KIR3DL20a
Mamu-KIR3DL20*010
Mamu-KIR3DL20*007
Mamu-KIR3DL20*003
Mamu-KIR3DL20*011
Mafa-KIR3DL20
Mamu-KIR3DL20*001
I
Gogo-KIR3DL7
KIR2DS4
KIR2DS5
KIR2DS3
KIR2DL1
KIR2DS1
Patr-KIR2DL6
Patr-KIR2DL8
KIR2DL2
KIR2DL3
KIR2DS2
III
0.05
B
D1
KIR3DL3*029
KIR3DL3*034
KIR3DL3*00202
KIR3DL3*00302
KIR3DL3*00601
Gogo-KIR3DL3
Patr-KIR3DL3*001
Papa-KIR3DL3
Patr-KIR3DL3*003
Patr-KIR3DL3*004
Poab-KIR3DL3*001
Popy-KIR3DL3*00402
Popy-KIR3DL3*003
Poab-KIR3DL3*005
Hoho-KIR3DL3 H2
Hoho-3DL3 H1
Nole-KIR3DL3 H1
Nole-KIR3DL3 H2
Paan-KIR3DL20a
Pite-KIR3dl3
Cogu-KIR3DL20
Chae-KIR3DL20
Mafa-KIR3DL20
Mamu-KIR3DL20*001
Mamu-KIR3DL20*010
Mamu-KIR3DL20*007
Mamu-KIR3DL20*003
Mamu-KIR3DL20*011
V
Lineage IV KIRs
IV
KIR3DL1*01501
KIR3DS1*01301
Patr-KIR3DL1*001
Popy-KIR3DL1*001
Poab-KIR3DS1*001
Poab-KIR3DL1*001
KIR3DL2*00101
Papa-KIR3DLc
Papa-KIR3DSa
II
Poab-KIR2DL11*001
Poab-KIR2DS13*001
Popy-KIR2DS13*001
Poab-KIR2DL12*001
Poab-KIR2DS10*001
Poab-KIR2DL10*001
Popy-KIR2DS10*001
Gogo-KIR2DL6
Poab-KIR2DS14*001
KIR2DL2*00101
KIR2DL3*00101
KIR2DS2*00101
Gogo-KIR2DSa
KIR2DS4*00101
Patr-KIR2DS4*001
Gogo-KIR2DLb
Gogo-KIR3DLa.v2
Gogo-KIR3DLa.v1
Patr-KIR2DL9*001
Gogo-KIR2DLd
Patr-KIR3DS6*001
Patr-KIR2DL6*001
Patr-KIR2DL8*001
Gogo-KIR2DLe
Patr-KIR3DL5*001
Gogo-KIR3DL7
Patr-KIR2DL7*001
Gogo-KIR2DLc
KIR2DS3*00101
KIR2DS5*001
KIR2DL1*001
KIR2DS1*001
Patr-KIR3DS2*001
Patr-KIR3DL4*001
Papa-KIR3DL4
III
0.020

Fig. S2 A/B

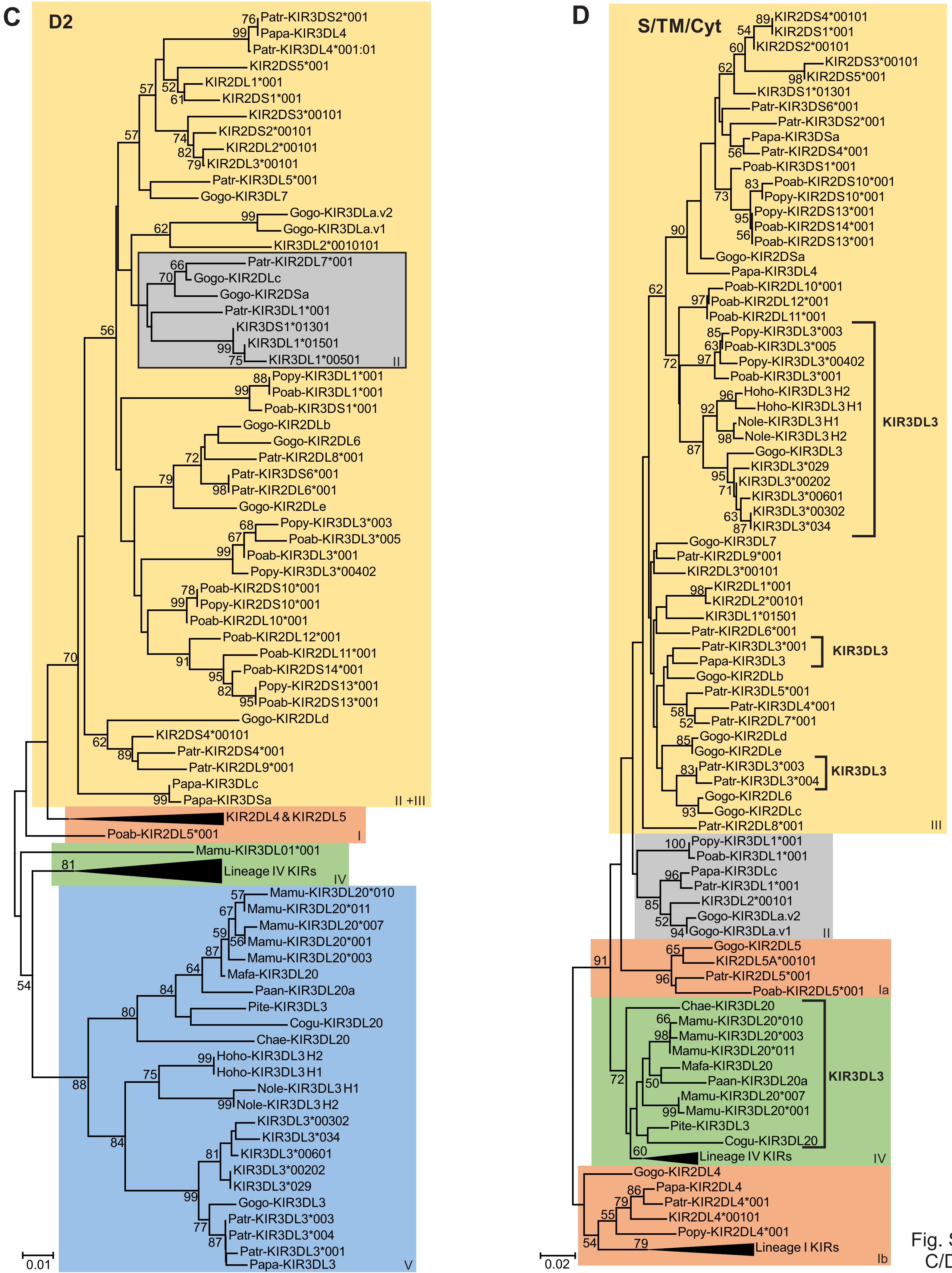
C
D2
Patr-KIR3DS2*001
Papa-KIR3DL4
Patr-KIR3DL4*001:01
KIR2DS5*001
KIR2DL1*001
KIR2DS1*001
KIR2DS3*00101
KIR2DS2*00101
KIR2DL2*00101
KIR2DL3*00101
Patr-KIR3DL5*001
Gogo-KIR3DL7
Gogo-KIR3DLa.v2
Gogo-KIR3DLa.v1
KIR3DL2*0010101
Patr-KIR2DL7*001
Gogo-KIR2DLc
Gogo-KIR2DSa
Patr-KIR3DL1*001
KIR3DS1*01301
KIR3DL1*01501
KIR3DL1*00501
II
Popy-KIR3DL1*001
Poab-KIR3DL1*001
Poab-KIR3DS1*001
Gogo-KIR2DLb
Gogo-KIR2DL6
Patr-KIR2DL8*001
Patr-KIR3DS6*001
Patr-KIR2DL6*001
Gogo-KIR2DLe
Popy-KIR3DL3*003
Poab-KIR3DL3*005
Poab-KIR3DL3*001
Popy-KIR3DL3*00402
Poab-KIR2DS10*001
Popy-KIR2DS10*001
Poab-KIR2DL10*001
Poab-KIR2DL12*001
Poab-KIR2DL11*001
Poab-KIR2DS14*001
Popy-KIR2DS13*001
Poab-KIR2DS13*001
Gogo-KIR2DLd
KIR2DS4*00101
Patr-KIR2DS4*001
Patr-KIR2DL9*001
Papa-KIR3DLc
Papa-KIR3DSa
II +III
KIR2DL4 & KIR2DL5
Poab-KIR2DL5*001
I
Mamu-KIR3DL01*001
Lineage IV KIRs
IV
Mamu-KIR3DL20*010
Mamu-KIR3DL20*011
Mamu-KIR3DL20*007
Mamu-KIR3DL20*001
Mamu-KIR3DL20*003
Mafa-KIR3DL20
Paan-KIR3DL20a
Pite-KIR3DL3
Cogu-KIR3DL20
Chae-KIR3DL20
Hoho-KIR3DL3 H2
Hoho-KIR3DL3 H1
Nole-KIR3DL3 H1
Nole-KIR3DL3 H2
KIR3DL3*00302
KIR3DL3*034
KIR3DL3*00601
KIR3DL3*00202
KIR3DL3*029
Gogo-KIR3DL3
Patr-KIR3DL3*003
Patr-KIR3DL3*004
Patr-KIR3DL3*001
Papa-KIR3DL3
V
0.01
D
S/TM/Cyt
KIR2DS4*00101
KIR2DS1*001
KIR2DS2*00101
KIR2DS3*00101
KIR2DS5*001
KIR3DS1*01301
Patr-KIR3DS6*001
Patr-KIR3DS2*001
Papa-KIR3DSa
Patr-KIR2DS4*001
Poab-KIR3DS1*001
Poab-KIR2DS10*001
Popy-KIR2DS10*001
Popy-KIR2DS13*001
Poab-KIR2DS14*001
Poab-KIR2DS13*001
Gogo-KIR2DSa
Papa-KIR3DL4
Poab-KIR2DL10*001
Poab-KIR2DL12*001
Poab-KIR2DL11*001
Popy-KIR3DL3*003
Poab-KIR3DL3*005
Popy-KIR3DL3*00402
Poab-KIR3DL3*001
Hoho-KIR3DL3 H2
Hoho-KIR3DL3 H1
Nole-KIR3DL3 H1
Nole-KIR3DL3 H2
Gogo-KIR3DL3
KIR3DL3*029
KIR3DL3*00202
KIR3DL3*00601
KIR3DL3*00302
KIR3DL3*034
KIR3DL3
Gogo-KIR3DL7
Patr-KIR2DL9*001
KIR2DL3*00101
KIR2DL1*001
KIR2DL2*00101
KIR3DL1*01501
Patr-KIR2DL6*001
Patr-KIR3DL3*001
Papa-KIR3DL3
KIR3DL3
Gogo-KIR2DLb
Patr-KIR3DL5*001
Patr-KIR3DL4*001
Patr-KIR2DL7*001
Gogo-KIR2DLd
Gogo-KIR2DLe
Patr-KIR3DL3*003
Patr-KIR3DL3*004
KIR3DL3
Gogo-KIR2DL6
Gogo-KIR2DLc
Patr-KIR2DL8*001
III
Popy-KIR3DL1*001
Poab-KIR3DL1*001
Papa-KIR3DLc
Patr-KIR3DL1*001
KIR3DL2*00101
Gogo-KIR3DLa.v2
Gogo-KIR3DLa.v1
II
Gogo-KIR2DL5
KIR2DL5A*00101
Patr-KIR2DL5*001
Poab-KIR2DL5*001
Ia
Chae-KIR3DL20
Mamu-KIR3DL20*010
Mamu-KIR3DL20*003
Mamu-KIR3DL20*011
Mafa-KIR3DL20
Paan-KIR3DL20a
Mamu-KIR3DL20*007
Mamu-KIR3DL20*001
Pite-KIR3DL3
Cogu-KIR3DL20
KIR3DL3
Lineage IV KIRs
IV
Gogo-KIR2DL4
Papa-KIR2DL4
Patr-KIR2DL4*001
KIR2DL4*00101
Popy-KIR2DL4*001
Lineage I KIRs
Ib
0.02

Fig. S2
C/D

	LP		D0											D1											D2											Tail							
	-19	-3	13*	15	17	31	34	35	45	56	79	82	86	111	113	122	128	131	143	145	147	160	168	195	198	210	226	233	260	269	274	277	282	287	292	300	303	310	326	327	352	361	374
KIR3DL3*001	S	G	W	G	V	R	F	N	M	R	P	P	S	P	V	Q	R	R	P	R	I	S	L	V	G	G	R	H	P	N	G	R	A	S	V	N	V	V	C	A	E	A	T
KIR3DL3*002	-	-	-	-	-	-	-	-	-	-	-	-	-	-	-	-	-	-	-	-	V	-	-	-	-	-	-	-	-	-	-	-	-	-	-	H	-	-	-	-	-	-	-
KIR3DL3*003	-	-	-	-	-	-	-	-	-	-	-	-	-	-	-	-	-	-	-	-	-	-	-	-	-	-	-	-	-	-	-	-	-	-	-	Y	A	-	-	-	-	-	-
KIR3DL3*004	-	-	-	-	-	H	-	-	-	-	-	-	-	-	-	-	-	-	-	-	V	-	-	-	-	-	-	-	-	-	-	-	-	-	-	Y	A	-	-	-	-	-	-
KIR3DL3*005	-	-	-	-	-	-	-	-	-	-	-	-	-	-	-	-	-	-	-	-	V	-	-	-	-	D	-	-	-	-	-	-	-	-	-	H	-	-	-	-	-	-	-
KIR3DL3*006	-	-	-	-	-	-	-	K	-	-	-	-	-	-	-	-	S	-	-	-	V	-	-	-	-	-	-	-	-	T	-	-	-	-	-	-	-	-	-	-	-	-	-
KIR3DL3*007	-	-	-	-	-	-	-	-	-	-	-	-	-	-	-	-	-	H	-	-	V	-	-	-	-	-	-	-	-	-	-	-	-	-	-	Y	A	-	-	-	-	-	-
KIR3DL3*008	-	-	-	-	-	H	-	-	-	-	-	-	-	-	-	-	-	-	-	-	V	-	-	-	-	-	-	-	-	-	-	-	-	-	-	H	-	-	-	-	-	-	-
KIR3DL3*009	-	-	-	-	-	-	-	-	-	-	-	-	-	-	-	-	-	-	-	-	-	-	-	-	-	-	-	-	-	-	-	-	-	-	-	H	-	-	-	-	-	-	-
KIR3DL3*010	-	-	-	-	-	-	-	-	-	W	-	-	-	-	-	-	-	-	-	-	-	-	-	-	-	-	-	-	-	-	-	-	-	-	-	H	-	-	-	-	-	-	-
KIR3DL3*011	-	-	-	-	-	-	-	-	-	-	-	-	-	-	-	-	-	-	-	-	V	-	-	-	-	-	-	-	-	T	-	-	-	-	-	H	-	-	-	-	-	-	-
KIR3DL3*012	-	-	-	-	-	-	-	K	-	-	-	-	-	-	-	-	-	-	-	-	V	-	-	-	-	-	-	-	-	T	-	-	-	-	-	H	-	-	-	-	-	-	-
KIR3DL3*012R	-	-	-	-	-	-	-	K	-	-	-	-	-	-	-	-	-	-	-	-	V	-	-	-	-	-	-	-	-	T	-	-	-	-	-	H	-	-	-	P	-	-	-
KIR3DL3*013	-	-	-	-	-	-	-	-	-	-	-	-	-	-	-	-	-	-	-	-	V	-	-	-	-	-	-	-	-	-	-	-	-	-	-	-	-	-	-	-	-	-	-
KIR3DL3_013H	-	-	-	-	-	-	-	-	-	-	-	-	-	-	-	-	-	-	-	-	V	-	-	-	-	-	-	-	-	-	-	H	-	-	-	-	-	-	-	-	-	-	-
KIR3DL3*014	-	-	-	-	-	-	-	-	-	-	-	-	-	-	-	-	-	-	-	-	V	-	-	-	-	-	-	-	-	-	-	-	-	-	-	Y	A	-	-	-	-	-	-
KIR3DL3_014F	-	-	-	-	-	-	-	-	-	-	-	-	-	-	-	-	-	-	-	-	V	-	F	-	-	-	-	-	-	-	-	-	-	-	-	Y	A	-	-	-	-	-	-
KIR3DL3_014FT	-	-	-	-	-	-	-	-	-	-	-	-	-	-	-	-	-	-	-	-	V	-	F	-	-	-	-	-	-	T	-	-	-	-	-	Y	A	-	-	-	-	-	-
KIR3DL3*015	-	-	-	-	-	-	-	K	-	-	-	-	-	-	-	-	S	-	-	-	V	-	-	-	-	-	-	-	-	T	-	-	-	-	-	H	-	-	-	-	-	-	-
KIR3DL3*016	-	-	-	-	-	-	-	K	-	-	-	-	-	-	-	-	S	-	-	-	V	-	-	-	-	-	-	-	-	T	-	-	-	-	-	Y	A	-	-	-	-	-	-
KIR3DL3*017	-	-	-	-	-	-	-	-	-	-	-	-	-	-	-	-	-	-	-	-	V	-	-	-	-	-	-	-	-	T	-	-	-	-	-	-	-	-	-	-	-	-	-
KIR3DL3*017R	-	-	-	-	-	-	-	-	-	Q	-	-	-	-	-	-	-	-	-	-	V	-	-	-	-	-	-	-	-	T	-	-	-	-	-	-	-	-	-	-	-	-	-
KIR3DL3*018	-	-	-	-	-	-	-	-	-	-	-	-	-	-	-	-	S	-	-	-	V	-	-	-	-	-	-	-	-	T	-	-	-	-	-	H	-	-	-	-	-	-	-
KIR3DL3*019	-	-	-	-	-	-	-	-	-	-	-	-	-	-	-	-	-	H	-	-	V	-	-	-	-	-	-	-	-	-	-	-	-	-	-	-	-	-	-	-	-	-	-
KIR3DL3*020	-	-	-	-	-	-	-	-	-	-	-	-	-	-	-	-	-	-	-	-	V	-	-	-	-	-	-	-	-	T	-	-	-	-	-	Y	A	-	-	-	-	-	-
KIR3DL3*021	-	-	-	-	-	-	-	-	-	-	-	-	-	-	-	-	S	-	-	-	V	-	-	-	-	-	-	-	-	T	-	-	-	-	-	-	-	-	-	-	-	-	-
KIR3DL3*022	-	-	-	-	-	-	-	K	-	-	-	-	-	-	-	-	-	-	-	-	V	-	-	-	-	-	-	-	-	T	-	-	-	-	-	Y	A	-	-	-	-	-	-
KIR3DL3*023	-	-	-	-	-	-	-	K	-	-	-	-	-	-	-	-	-	-	-	-	-	-	-	-	-	-	-	-	-	-	-	-	-	-	-	H	-	-	-	-	-	-	-
KIR3DL3*025	-	-	-	-	-	-	-	K	-	-	-	-	-	-	-	-	S	-	-	-	V	-	-	-	-	-	-	-	-	T	-	-	-	-	-	H	-	-	-	P	D	-	-
KIR3DL3*026	-	-	-	-	-	H	-	-	-	-	-	-	-	-	-	-	-	-	-	-	V	-	-	-	-	-	-	-	-	-	-	-	-	-	-	-	-	-	-	-	-	-	-
KIR3DL3*027	-	-	-	-	-	-	-	-	-	-	-	-	-	-	-	-	-	-	-	-	V	-	-	-	-	-	-	-	-	-	-	-	-	-	-	H	-	-	-	P	D	-	-
KIR3DL3*028	-	-	-	-	-	-	-	-	-	W	-	-	-	-	-	-	-	-	-	-	-	-	-	-	-	-	-	-	-	-	-	-	-	-	-	Y	A	-	-	-	-	-	-
KIR3DL3*029	-	-	-	-	-	-	-	-	-	-	-	-	-	-	-	-	-	-	-	-	V	-	-	-	-	-	-	-	-	-	-	-	-	-	-	H	D	-	-	P	D	-	-
KIR3DL3*030	-	-	-	-	-	-	-	-	-	-	-	-	-	-	-	-	-	H	-	-	V	-	-	-	-	-	-	-	-	-	-	-	-	-	-	H	-	-	-	-	-	-	-
KIR3DL3*032	-	-	-	-	-	-	-	-	-	-	-	-	-	L	-	-	-	-	-	-	-	-	-	-	-	-	-	-	-	-	-	-	-	-	-	Y	A	-	-	-	-	-	-
KIR3DL3*033	-	-	-	-	-	H	-	-	K	-	-	-	-	-	-	-	-	-	-	-	V	-	-	-	-	-	-	-	-	-	-	-	-	-	-	Y	A	-	-	-	-	-	-
KIR3DL3*034	-	-	-	-	-	-	-	-	-	-	-	-	-	-	-	-	-	-	-	-	V	-	-	-	-	D	-	-	-	-	-	-	-	-	-	Y	A	-	-	-	-	-	-
KIR3DL3*036	-	-	-	-	-	-	-	-	-	-	-	-	-	-	-	-	S	-	-	-	V	-	-	-	-	-	-	-	-	T	-	-	-	-	-	Y	A	-	-	-	-	-	-
KIR3DL3*037	-	-	-	-	-	-	-	-	-	-	-	-	-	-	-	-	-	-	-	C	-	-	-	-	-	-	-	-	-	-	-	-	-	-	-	Y	A	-	-	-	-	-	-
KIR3DL3*038	-	-	-	-	-	-	-	-	-	-	-	-	-	-	-	-	-	-	-	-	V	-	-	A	-	-	-	-	-	T	-	-	-	-	-	Y	A	-	-	-	-	-	-
KIR3DL3*039	-	-	-	-	-	-	-	-	-	-	-	-	P	-	-	-	-	-	-	-	V	-	-	-	-	-	-	-	-	-	-	-	-	-	-	Y	A	-	-	-	-	-	-
KIR3DL3*040	-	-	-	-	-	-	-	-	-	-	-	-	-	-	-	-	-	-	-	-	V	-	-	-	-	-	-	-	-	-	-	-	-	-	I	H	-	-	-	P	D	-	-
KIR3DL3*041	-	-	-	-	-	-	-	-	-	-	-	-	-	-	-	-	-	-	-	-	-	-	-	-	-	-	-	-	-	-	-	-	-	-	-	H	-	-	-	P	D	-	-
KIR3DL3*042	-	-	-	-	-	-	-	-	-	-	-	-	-	-	-	-	-	-	-	-	V	-	-	-	-	D	-	-	-	-	-	-	-	-	-	-	-	-	-	-	-	-	-
KIR3DL3*043	-	-	-	-	-	-	-	-	-	-	A	-	-	-	-	-	-	-	-	-	-	-	-	-	-	-	-	-	-	-	-	-	-	-	-	H	-	-	-	-	-	-	-
KIR3DL3*044	-	-	-	-	-	-	-	-	-	-	-	-	-	-	-	-	-	-	-	-	-	-	-	-	D	-	-	-	-	-	-	-	-	-	-	H	-	-	-	-	-	-	-
KIR3DL3*045	-	-	-	-	-	-	-	-	-	-	-	-	-	-	-	-	-	-	-	-	-	-	-	-	-	-	-	-	-	-	-	-	-	R	-	Y	A	-	-	-	-	-	-
KIR3DL3*046	-	-	-	-	-	-	-	-	-	-	-	-	-	-	-	-	-	-	-	H	-	-	-	-	-	-	-	-	-	-	-	-	-	-	-	-	-	-	-	-	-	-	-
KIR3DL3*047	-	-	-	-	M	-	-	-	-	-	-	-	-	-	-	-	-	-	-	-	-	-	-	-	-	-	-	-	-	-	-	-	-	-	-	Y	A	-	-	-	-	-	-
KIR3DL3*048	-	-	-	-	-	-	-	-	-	W	-	-	-	-	-	-	-	-	-	-	-	-	-	-	-	-	-	-	-	-	-	-	-	-	-	-	-	-	-	-	-	-	-
KIR3DL3*049	-	-	-	-	-	H	-	-	-	-	-	-	-	-	-	-	-	-	-	-	V	-	-	-	-	-	-	-	-	-	-	-	-	-	-	H	-	-	-	P	D	-	-
KIR3DL3*049R	-	-	-	-	-	H	-	-	-	-	-	-	-	-	-	-	-	-	-	-	V	-	-	-	-	-	-	-	-	T	-	-	-	-	-	H	-	-	-	P	D	-	-
KIR3DL3*050	-	-	-	-	-	-	-	-	-	-	-	-	-	-	-	-	-	-	-	-	V	-	-	-	-	-	-	-	-	-	-	-	-	-	I	Y	A	-	-	-	-	-	-
KIR3DL3*051	-	-	-	-	-	-	-	K	-	-	-	-	-	-	-	-	-	-	-	-	V	-	-	-	-	-	-	-	-	-	-	-	-	-	-	H	-	-	-	-	-	-	-
KIR3DL3*052	-	-	-	-	-	-	-	-	-	-	-	-	P	-	-	-	-	-	-	-	-	-	-	-	-	-	-	-	-	-	-	-	-	-	-	H	-	-	-	-	-	-	-
KIR3DL3*053	-	-	-	-	-	-	-	-	-	-	-	-	-	-	-	-	-	-	-	-	V	-	-	-	-	-	-	-	-	-	-	-	-	-	I	H	-	-	-	-	-	-	-
KIR3DL3*054	-	-	-	-	-	-	-	-	-	-	-	-	-	-	M	-	-	-	-	-	-	-	-	-	-	-	-	-	-	-	-	-	-	-	-	H	-	-	-	-	D	-	-
KIR3DL3*055	-	-	-	-	-	-	-	-	-	Q	-	-	-	-	-	-	-	-	-	-	V	-	-	-	-	D	-	-	-	-	-	-	-	-	-	H	-	-	-	-	-	-	-
KIR3DL3*056	-	-	-	-	-	-	-	-	-	-	-	-	-	-	-	-	-	-	-	-	V	-	-	-	-	-	-	-	-	T	-	-	-	-	-	H	-	-	-	P	D	-	-
KIR3DL3*057	-	-	-	-	-	H	-	-	-	-	-	-	-	-	-	-	-	-	-	-	V	-	-	-	-	-	-	-	-	T	-	-	-	-	-	H	-	-	-	-	-	-	-
KIR3DL3*058	-	-	-	-	-	-	-	-	-	-	-	-	-	-	-	R	-	-	-	-	V	-	-	-	-	-	-	-	-	T	-	-	-	-	-	H	-	-	-	P	D	-	-
KIR3DL3*059	-	-	-	-	-	-	-	-	-	-	-	-	P	-	-	-	-	-	-	-	-	-	-	-	-	-	-	-	-	-	-	-	-	-	-	-	-	-	-	-	D	-	-
KIR3DL3*060	-	-	-	-	-	-	-	-	-	W	-	-	-	-	-	-	-	-	-	-	-	-	-	-	-	-	-	-	-	-	-	-	-	-	-	H	-	-	-	-	-	-	I
KIR3DL3*061	-	-	-	-	-	-	-	-	-	-	-	-	-	L	-	-	-	-	-	-	-	-	-	-	-	-	-	-	-	-	-	-	-	-	-	H	-	-	-	-	-	-	I
KIR3DL3*062	-	-	-	-	-	H	-	-	-	-	-	-	-	-	-	-	-	-	-	-	V	-	-	-	-	-	-	-	-	-	-	-	-	-	-	-	-	-	-	-	-	-	I
KIR3DL3*063	-	-	-	-	-	-	-	-	-	W	-	-	-	-	-	-	-	-	-	-	-	-	-	-	-	-	-	-	-	-	-	-	-	-	-	-	-	-	-	-	-	-	I
KIR3DL3*064	-	-	-	-	-	-	-	K	-	-	-	-	-	-	-	-	S	-	-	-	V	-	-	-	-	-	-	-	-	T	-	-	-	-	-	-	-	-	-	-	-	-	-
KIR3DL3*065	-	-	-	-	-	-	-	-	-	-	-	-	-	-	-	-	-	-	-	-	-	-	-	-	-	-	-	-	-	-	-	-	-	-	-	-	-	-	-	-	-	-	-
KIR3DL3*066	-	-	-	-	-	H	-	-	-	-	-	-	-	-	-	-	-	-	-	-	-	-	-	-	-	-	-	-	-	-	-	-	-	-	-	-	-	-	-	-	-	-	-
KIR3DL3*067	-	-	-	-	-	-	-	-	-	-	-	-	-	-	-	-	-	-	-	-	V	-	F	-	-	-	-	-	-	T	-	-	-	-	-	H	-	-	-	-	-	-	-
KIR3DL3*068	-	-	-	-	-	-	-	-	-	Q	-	-	-	-	-	-	-	-	-	-	V	T	F	-	-	-	-	-	-	T	-	-	-	-	-	H	-	-	-	-	-	-	-
KIR3DL3*069	-	-	-	-	-	-	-	-	-	-	-	-	P	-	-	-	-	-	-	-	V	-	-	-	-	-	-	-	-	T	-	-	-	-	-	Y	A	-	-	-	D	-	-
KIR3DL3*070	-	-	-	S	-	-	-	-	-	-	-	-	-	-	-	-	-	-	-	-	V	-	-	-	-	-	-	-	-	-	-	-	-	-	-	Y	A	-	-	-	-	-	-
KIR3DL3*075	-	-	-	-	-	-	-	-	-	-	-	-	-	-	-	-	-	-	-	-	V	-	-	-	-	-	-	-	-	-	-	-	-	-	-	H	-	-	-	-	-	-	-
KIR3DL3*077	-	-	-	-	-	-	-	-	-	-	-	-	-	-	-	-	-	-	H	-	-	-	-	-	-	-	-	-	-	-	-	-	-	-	-	-	-	-	-	-	-	-	-
KIR3DL3*078	-	-	-	-	-	-	-	-	-	-	-	-	-	-	-	-	-	-	-	-	-	-	-	-	-	-	-	-	-	-	-	-	-	-	-	-	-	L	-	-	-	-	-
KIR3DL3*079	-	-	-	-	-	-	-	-	-	-	-	-	-	-	-	-	-	-	-	-	-	F	-	-	-	-	-	-	-	-	-	-	-	-	-	Y	A	-	-	-	-	-	-
KIR3DL3*080N	-	-	*																																						-	-	-
KIR3DL3*081	L	-	-	-	-	-	-	-	-	-	-	-	-	-	-	-	-	-	-	-	-	-	-	-	-	-	-	-	-	-	-	-	-	-	-	H	-	-	-	-			
KIR3DL3*082	-	-	-	-	-	-	-	K	-	-	-	-	-	-	-	-	S	-	-	-	V	-	-	-	-	-	-	-	-	T	-	-	-	-	-	H	-	-	-	H	-	-	-
KIR3DL3*083	-	-	-	-	-	-	-	-	-	-	-	-	-	-	-	-	-	-	-	S	V	-	-	-	-	-	-	-	-	-	-	-	-	-	-	Y	A	-	-	-	-	T	-
KIR3DL3*085	-	-	-	-	-	-	-	-	-	-	-	-	-	-	-	-	-	-	-	H	-	-	-	-	-	-	-	-	-	-	-	-	-	-	-	H	-	-	-	-	-	-	-
KIR3DL3*086	-	-	-	-	-	-	-	-	-	-	-	-	-	-	-	-	-	-	-	-	V	-	-	-	-	-	Q	-	-	-	-	-	-	-	-	Y	A	-	-	-	-	-	-
KIR3DL3*087	-	-	-	-	-	-	-	-	-	-	-	-	-	-	-	-	-	-	-	-	-	-	-	-	-	-	-	R	-	-	-	-	-	-	-	Y	A	-	-	-	-	-	-
KIR3DL3*088	-	-	-	-	-	-	-	-	-	-	-	-	-	-	-	-	-	-	-	-	-	-	-	-	-	-	-	-	A	-	-	-	-	-	-	H	-	-	-	-	-	-	-
KIR3DL3*089	-	-	-	-	-	-	-	-	-	-	-	-	-	-	-	-	-	-	-	-	V	-	-	-	-	-	-	-	-	-	-	-	-	-	-	Y	A	-	Y	-	-	-	-
KIR3DL3*090	-	-	-	-	-	-	-	-	-	-	-	-	-	-	-	-	-	-	-	-	-	-	-	-	-	-	-	-	-	-	-	-	T	-	-	Y	A	-	-	-	-	-	-
KIR3DL3*091	-	-	-	-	-	-	-	-	-	-	-	L	-	-	-	-	-	-	-	-	-	-	-	-	-	-	-	-	-	-	-	-	-	-	-	Y	A	-	-	-	-	-	-
KIR3DL3*094	-	-	-	-	-	-	L	-	-	-	-	L	-	-	-	-	-	-	-	-	-	-	-	-	-	-	-	-	-	-	-	-	-	-	-	Y	A	-	-	-	-	-	-

Fig. S3

		Nucleotide		Amino Acid		
Population	N	pos^	change	pos^	change	Domain
ITU	1	5	C < T	-19	Ser > Leu	Leader
LWK	1	16	G > A	-15	Val > Ile	
LWK	1	36	G > T	-9	syn	
STU	1	216	C > T	51	syn	D0
ITU	1	302	A > G	80	His > Arg	
GWD	1	311	C > T	83	Thr > Ile	
STU	1	330	C > G	89	Ser > Arg	
GWD	1	393	T > C	110	syn	D1
LWK	1	393	T >G	110	syn	
PJL	1	406	T > C	115	Lys > Pro	
GWD,JPT	2	416	C > T	118	Val > Met	
TSI	1	434	G > C	124	Trp > Ser	
GIH	1	503	T > C	147	Ile > Thr	
PJL	1	517	G > A	152	Asp > Asn	
GIH	1	663	T > C	200	syn	D2
ITU	1	690	G > A	209	syn	
CHS	1	724	T > G	221	Leu > Val	
CDX	1	737	C > T	225	Ser > Phe	
IBS	1	750	T > G	229	Phe > Leu	
CHS,JPT	3	775	G > A	238	Ala > Thr	
CHS	1	803	C > A	247	Ala > Glu	
MSL	1	878	G > A	272	Cys > Tyr	
PEL	1	881	T > A	273	Phe > Tyr	
PEL	1	902	C > G	280	Pro > Arg	
CHS, CDX, KHV	3	916	G > C	285	Asp > His	
ACB	1	1154	A > G	364	Ans > Ser	Cyt
CHS, CDX, KHV, ITU	9	1184	C < T	374	Thr > Ile	
CHB	1	1228	G < A	389	Val > Met	

Fig. S4

	Gha	Han	Eur	Kho	Maori	Nama	PPNG	Yuc
KIR3DL3	0.94	0.75	0.95	0.91	0.92	0.95	0.81	0.67
KIR3DL1	0.92	0.71	0.88	0.93	0.88	0.94	0.68	0.65
KIR3DL2	0.90	0.69	0.89	0.93	0.75	0.93	0.57	0.55
KIR2DL1	0.81	0.44	0.76	0.87	0.59	0.85	n/a	0.51
KIR2DL2/3	0.77	0.48	0.76	0.83	0.63	0.84	n/a	0.63
HLA-A	0.92	0.85	0.89	0.95	0.78	0.96	0.43	0.68
HLA-B	0.93	0.93	0.96	0.95	0.83	0.94	0.86	0.70
HLA-C	0.88	0.89	0.92	0.90	0.77	0.93	0.75	0.42

Fig. S5

A

	Residue	P=	AA	Var
D0	56	0.98	R	W, Q
D1	145	0.97	R	C, H, S
	147	0.97	I	V
D2	199	0.92	L	V
	246	0.95	T	P
	267	0.95	G	E, A
	282	0.96	A	V, T
S/TM/Cyt	301	0.91	Y	H, Q, N
	311	0.91	V	I, L
	328	0.90	A	S, P, H
	375	0.93	T	I
	376	0.90	R	H, P, C
	389	0.97	S	R, I

B

Species	Residue 145	147
Human	Arg / His / Cys	Val / Iso / (Thr)
Chimpanzee	Arg	Val / Iso
Orangutan	Arg	Val
Gorilla	Arg	Val
Gibbon	Cys	Val

Fig. S6
